# Diagnostic Value of Whole-Body MRI in Pediatric Patients with Suspected Rheumatic Diseases

**DOI:** 10.3390/medicina60091407

**Published:** 2024-08-28

**Authors:** Joanna Ożga, Monika Ostrogórska, Wadim Wojciechowski, Zbigniew Żuber

**Affiliations:** 1 Department of Pediatrics, Faculty of Medicine and Health Sciences, Andrzej Frycz Modrzewski Krakow University, 30-705 Krakow, Poland; 2 Clinical Department of Pediatrics and Rheumatology, St. Louis Regional Specialised Children’s Hospital, 31-034 Krakow, Poland; 3 Department of Radiology, Jagiellonian University Medical College, 31-503 Krakow, Poland

**Keywords:** whole-body MRI, juvenile idiopathic arthritis, juvenile idiopathic inflammatory myopathies, chronic nonbacterial osteomyelitis

## Abstract

*Background and Objectives*: The diagnosis of rheumatic diseases in children is challenging and requires the use of advanced imaging examinations such as whole-body magnetic resonance imaging (MRI). Whole-body MRI allows visualization of bone marrow edema (BME), muscle edema, joint effusion and changes in the soft tissues surrounding the joints. The aim of this study was to collect and compare whole-body MRI findings, laboratory results and clinical manifestations of pediatric patients with suspected rheumatic disease. *Materials and methods*: In this retrospective single-center study, 33 patients who underwent whole-body MRI were included. Their age ranged from 9 to 17 years, and 24 (72.73%) of the patients were female. Patients were diagnosed as follows: juvenile idiopathic arthritis (27.27%), juvenile idiopathic inflammatory myopathies (21.21%), chronic nonbacterial osteomyelitis (21.21%) and other medical conditions (30.30%), such as arthritis associated with infection, scleroderma, Takayasu arteritis, polyarteritis nodosa and joint damage. *Results*: The most common symptom reported by 26 (79.79%) patients was pain. On physical examination, the limitation of joint mobility was examined in 17 (51.51%), swelling of the joints was observed in 12 (36.36%) patients and decreased muscle strength was noticed in 11 (33.33%) patients. An increase in the C-reactive protein (12%), erythrocyte sedimentation rate (9%), leukocyte count (9%) and creatine kinase (CK) (18%) was observed. Whole-body MRI revealed myositis (30%), joint effusion (27%) and BME (24%). The statistical analysis showed a significant relationship between myositis and the elevated CK level (*p* < 0.05). *Conclusions*: The most common symptom in the studied population was pain, while the limitation of joint mobility was found in more than half of patients. Myositis was the most commonly imaged lesion on the whole-body MRI and it was related to an increase in the CK level.

## 1. Introduction

Whole-body magnetic resonance imaging (MRI) is an examination that is used to visualize the entire body in one scan while not using any ionizing radiation. There is no standard whole-body MRI protocol, as specific sequences are selected depending on the specific indications [1]. The T1-weighted, T2-weighted, and short tau inversion recovery/turbo inversion recovery magnitude (STIR/TIRM) sequences are used for establishing the diagnosis of pathological changes in the synovial membrane, subchondral bone marrow, and surrounding soft tissues [2,3]. 

This method’s application includes diagnosis and monitoring of rheumatic diseases in children, such as juvenile idiopathic arthritis (JIA) [4], juvenile idiopathic inflammatory myopathies (JIIMs) [5], and chronic nonbacterial osteomyelitis (CNO) [6], as well as detection of skeletal metastases of malignant tumors in children [7]. 

JIA is an autoimmune, non-infectious, inflammatory joint disease of unknown etiology that begins before the age of 16, with symptoms lasting at least six weeks [8]. It is the most common rheumatic disease in children, with an estimated prevalence of 1 per 1000 children and an incidence of 2 to 28 per 100,000 pediatric population compared to nearly 10 per 100,000 pediatric population in Poland [9]. The main symptoms are non-specific and consist of chronic pain, limitation of motion and swelling of joints, mobility difficulties, recurrent fever, fatigue, weight loss and growth failure [10]. Laboratory tests performed in patients with suspicion of JIA include the complete blood count (CBC), erythrocyte sedimentation rate (ESR), C-reactive protein (CRP), antinuclear antibody test (ANA), rheumatoid factor (RF), human leukocyte antigen B27 (HLA-B27) and human leukocyte antigen Cw6 (HLA-Cw6) [11]. Each patient should also undergo essential blood tests, including the electrolytes levels, parameters to assess liver and kidney function, such as aspartate transaminase (AST), alanine transaminase (ALT) [12] and gamma-glutamyl transpeptidase (GGTP), and creatinine [13]. Imaging procedures used to diagnose and monitor the course of JIA are ultrasonography (US), X-ray, magnetic resonance imaging (MRI), computed tomography (CT), scintigraphy and positron emission tomography [14]. MRI of the musculoskeletal system has proved to be the most sensitive imaging method for diagnosis and identifying the type of JIA. It is also used to monitor the effectiveness of treatment of JIA [15], which includes nonsteroidal anti-inflammatory drugs (NSAIDs), glucocorticosteroids, and conventional and biologic disease-modifying drugs [16]. 

Juvenile idiopathic inflammatory myopathies (JIIMs) are heterogenous autoinflammatory diseases mainly affecting muscles and skin [17]. Two major clinicopathologic groups of JIIMs are juvenile dermatomyositis (JDM) and juvenile polymyositis (JPM) [18]. The estimated incidence is 1.6–4 cases per 1,000,000 children per year [19], with a female predominance (3:1). JIIMs are characterized by weakness in proximal muscles and pathognomonic skin rashes [20], with the presence of non-specific symptoms such as muscle pain, mobility difficulties, fatigue, weight loss and recurrent fever. The hallmark cutaneous signs of DM are Gottron’s papules, Gottron’s sign, heliotrope rash and V-neck sign [21,22]. The relevant laboratory tests are the CBC, ESR, CRP, thyroid-stimulating hormone (TSH), electrocytes, creatinine, lactate dehydrogenase (LDH), AST, ALT, creatine kinase (CK) and aldolase levels. In addition, a fasting glucose and lipid panel should be obtained to determine the risk of diabetes or hyperlipidemia [23]. The classic criteria for the diagnosis of JDM were modified in 2013 [23], establishing MRI as the recommended diagnostic method for JIIMs, as it allows accurate assessment of soft tissue abnormalities [24,25]. The typical lesions present on the STIR sequence of MRI include edema regions with increased signal intensity, which correlate with myositis of the fascia and subcutaneous tissue [26]. Whole-body MRI provides complete information about the extent of the disease activity [27] and is used to evaluate the response to treatment and possible adverse events such as sterile necrosis or fracture [28]. The pharmacological therapy for JIIMs consists of high-dose corticosteroids, initially in combination with disease-modifying drugs like methotrexate (MTX) or cyclosporin A (CsA) [29]. 

Chronic nonbacterial osteomyelitis (CNO) is an autoinflammatory bone disorder affecting children and adolescents, with the peak onset of between 7 years and 12 years of age and a female predominance [30,31,32]. Its prevalence is estimated at about 0.5–6 cases per 1,000,000 children [33]. 

Clinically, CNO manifests as bone pain, fatigue, local swelling, rarely redness and warming of the skin, associated skin symptoms (including palmoplantar pustulosis, psoriasis and acne), sometimes mildly elevated temperature and pathological fractures (usually of the affected bones) [33]. Blood inflammatory parameters, along with alkaline phosphatase (ALP) and lactone dehydrogenase (LDH), may be slightly elevated [34]. Both the Bristol and Jansson criteria include increased CRP and ESR level as well as MRI with STIR sequences showing bone marrow edema, bone proliferation, lytic areas and periosteal reaction [35,36]. Whole-body MRI in children with suspected CNO is considered to be the most sensitive imaging method as it reveals the multifocal pattern of disease as well as silent (nonpainful) lesions [37]. Follow-up whole-body MRI is commonly performed in order to verify if the patient needs extended pharmacological treatment, which involves NSAIDs, corticosteroids, sulfasalazine, methotrexate, TNF (tumor necrosis factor) inhibitors and bisphosphonates [38,39]. 

Whole-body MRI is one of the imaging examinations performed in patients with suspicion of rheumatic disease. Even though it is not widely available, this imaging method may be used in all patients regardless of their age and allows clinicians to determine the valid diagnosis and monitor the disease activity. Whole-body MRI has proved to be a method of choice in the diagnosis of JIIMs and CNO, as well as in the diagnosis of neoplastic processes. The aim of this study is to collect and compare the clinical symptoms, laboratory test results and whole-body MRI findings in pediatric patients hospitalized due to suspected rheumatic disease. 

## 2. Materials and Methods

### 2.1. Patients

This single-center retrospective study was performed in accordance with the Declaration of Helsinki and received approval from the Institutional Bioethics Committee (no. of approval: KBKA/52/O/2023, date of approval: 21 September 2023). In the study, 33 patients hospitalized in the department of pediatric rheumatology in Kraków, Poland, between December 2021 and April 2024 were included. The analysis was based on data from the hospital’s database and consisted of whole-body MRI, clinical observations, results of the laboratory tests and final diagnoses. All the patients underwent initial whole-body MRI due to suspected rheumatic disease. Patients were diagnosed with JIA, JIIM, CNO, arthritis associated with infection, scleroderma, Takayasu arteritis, polyarteritis nodosa and joint damage. 

### 2.2. Laboratory Tests 

Patients underwent basic laboratory tests, including CBC, glucose, urea, electrolytes, and inflammatory parameters such as ESR and CRP, as well as the following tests assessing liver and kidney function: AST, ALT, GGTP, ALP, creatinine. Additional blood tests covered the levels of enzymes associated with muscle function, such as LDH and CK, along with tests for rheumatic diseases: ANA, RF, HLA-B27 and HLA-Cw6 presence.

### 2.3. MRI Protocol and Interpretation of MRI Findings

All the whole-body MRI examinations were performed on a 3.0 Tesla MRI scanner (Achieva, Philips Healthcare, Amsterdam, the Netherlands) with an 8-channel phased-array XL-torso body matrix coil. The whole-body institutional protocol developed for the assessment of rheumatic diseases in the pediatric population was used. 

The position of the patient during the examination was as follows: the patient was supine, with the upper limbs positioned alongside the body. The patient remained in one position with free breathing during the whole procedure. Each examination lasted 30–40 min, depending on the patient’s co-operation. Sequential imaging was obtained in the frontal plane in the T1FFE (T1-weighted gradient echo), T2TSE (T2-weighted ultra-fast spin echo) and STIR sequences. 

Each examination was evaluated by a radiologist specializing in musculoskeletal MRI assessment who had more than 20 years of experience. The assessment included the following lesions: bone marrow edema (BME), myositis and the presence of synovial fluid, enlargement of organs such as the liver and spleen, and intraosseous ganglia. 

### 2.4. Statistical Analysis

All the data were analyzed using Statistica 13.3 software (StatSoft Inc., Krakow, Poland). Descriptive statistics methods were used for all the obtained laboratory test results and MRI results. The qualitative statistics included a chi-squared test (χ^2^ tests) for comparison between the presence of myositis or BME on MRI and the diagnosis, positive result for HLA-B27, HLA-Cw6, and ANA and the presence of fever or spine pain. Where possible, the MRI findings and symptoms were compared between diagnoses with the chi-squared test. The Mann–Whitney U test was used for comparison of the CK/LDH/leukocytes/CRP level results in patients regarding the presence of BME. The diagnosis and patients’ age were examined with one-way ANOVA. Two-way ANOVA was used for comparison between the leukocytes/CRP levels and both the presence of myositis or BME on MRI and the diagnosis. Post hoc Bonferroni correction was used if the ANOVA results were statistically significant. The statistical significance for each analysis was defined as *p* < 0.05.

## 3. Results

### 3.1. Demographic Data 

In the study group, 27.27% (*n* = 9) of patients were male, while 72.73% (*n* = 24) of patients were female. All the patients were divided into four groups based on their diagnosis: JIA—9 (27.27%) patients, JIIM—7 (21.21%) patients, CNO—7 (21.21%) patients and other medical conditions—10 (30.30%) patients. The age distribution of the study population is presented in Table 1. According to the one-way ANOVA, there were no statistically significant differences in the patients’ age between the four diagnoses.

### 3.2. Symptoms

**Figure 1:**
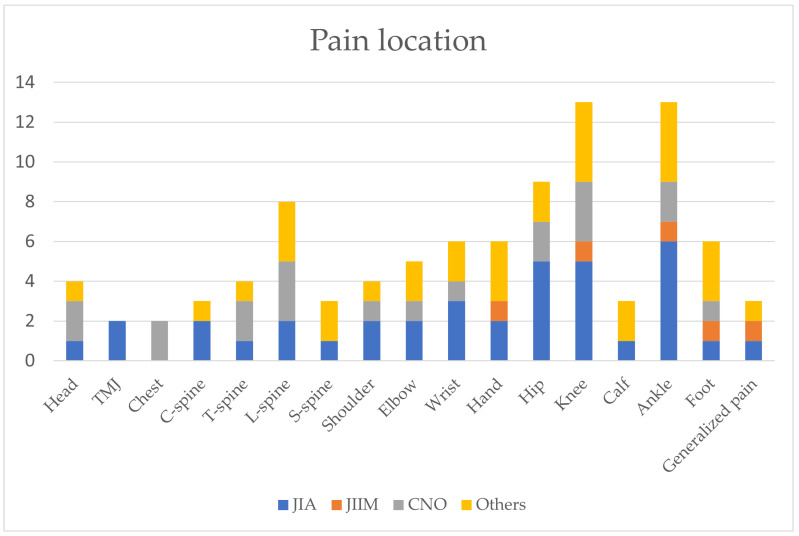
Graph presents the location of the pain reported by patients. TMJ—temporomandibular joint. C-spine—cervical spine. T-spine—thoracic spine. L-spine—lumbar spine. S-spine—sacral spine.

Pain was reported by 26 (79.79%) patients, with the locations depicted in Figure 1. No statistically significant differences were found in the frequency of individual symptoms for the different diagnoses.

Morning stiffness lasting more than an hour and resolving after movement was reported by five (15%) patients. Generalized fatigue was present in seven (21%) patients, while weight loss was recorded in three (9%) patients. On physical examination, decreased muscle strength was noticed in 11 (33%) subjects, of whom 3 (9%) also presented hypotonia. 

Skin lesions were present in 17 (51%) patients and included erythema on the face or neck in 6 patients (18%), psoriatic lesions in 4 (12%) patients, acne in 4 patients (12%) and cafe au lait macules in 2 patients (6%). Gottron’s sign was observed in two (6%) patients with JIIM, while Gottron’s papules occurred in two (6%) other patients with JIIM. 

In the study population, eight (24.24%) patients developed fever, defined as an elevation of body temperature above 38 degrees Celsius that occurred during at least two consecutive days. Among the patients with BME on MRI, fever occurred in three (37.50%) cases, while in patients with myositis on MRI, it occurred in one (10%) case. There was no significant statistical relationship between either BME or myositis on MRI and the presence of fever in patients (*p* > 0.05). 

### 3.3. Laboratory Findings

The inflammatory parameters were increased in four (12%) patients. Elevated CRP was observed in four (12%) patients, while elevated ER and leukocytosis were noted in three (9%) patients. The other CBC results in all the patients were within the reference values for age and gender. Elevation of liver function tests such as ALT occurred in five (15%) patients and AST in four (12%) patients. ALP was decreased in seven (21%) patients. CK was elevated in six (18%) patients. The glucose, urea, electrolytes, GGTP, LDH levels and RF were within the reference values for age in all the patients. 

The positive results for ANA, HLA-B27 and HLA-Cw-6 are presented in Table 2.

Among eight patients with BME on MRI, two (25%) were HLA-B27 positive; however, there was no statistical significance in the statistical comparison between the presence of BME and a positive result for HLA-B27 (*p* > 0.05). 

A statistically significantly elevated CK level was found for a diagnosis of JIIM (*p* < 0.05). No statistically significant relationship concerning the presence of BME and leukocytes or the CRP level was found in the Mann–Whitney U test. No statistically significant relationship was found for these data in the two-way ANOVA when diagnosis was additionally taken into account.

### 3.4. MRI Findings

The MRI findings are presented in Table 3. 

**Figure 2:**
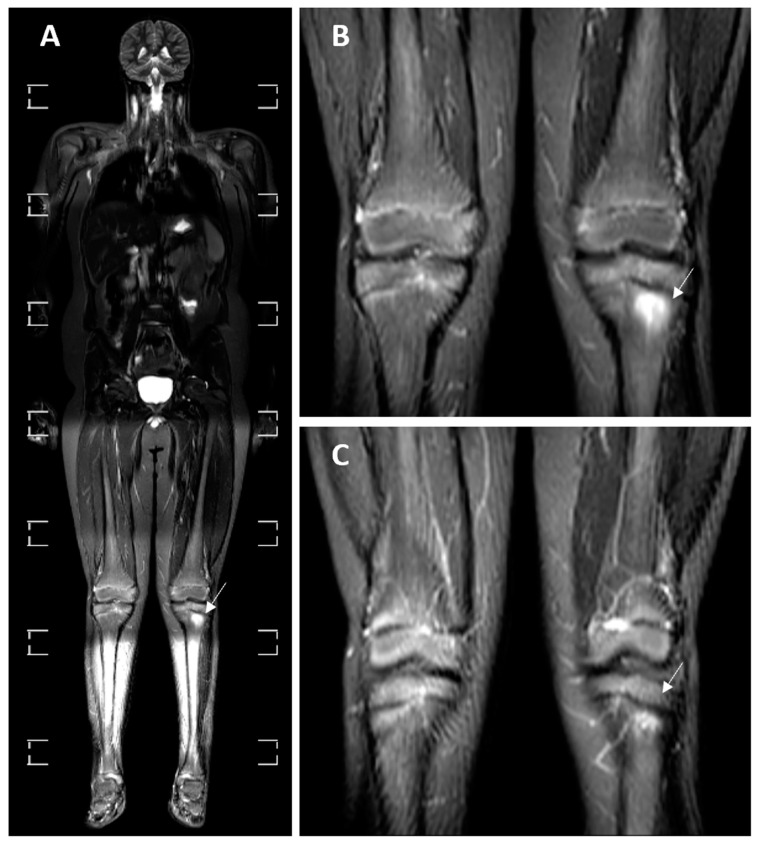
Whole-body MRI in the STIR sequence of an 11-year-old male patient with CNO and a history of joint pain. (**A**) A whole-body MRI scan. (**B**,**C**) Two consecutive cross-sections of the knee joints. The signal elevation in the left tibia in the epiphyseal and metaepiphyseal zones is notable, pointed by white arrows (**A**–**C**).

BME was visualized on eight (24%) MRI examinations (Figure 2). On three (37.50%) of them, a single inflammatory lesion was observed, while on the remaining five (62.50%), multifocal inflammation was described. BME was localized in the upper limbs on five (62.50%) examinations, in the lower limbs on three (37.50%) examinations and in the sacroiliac joints on two (25%) examinations. 

**Figure 3:**
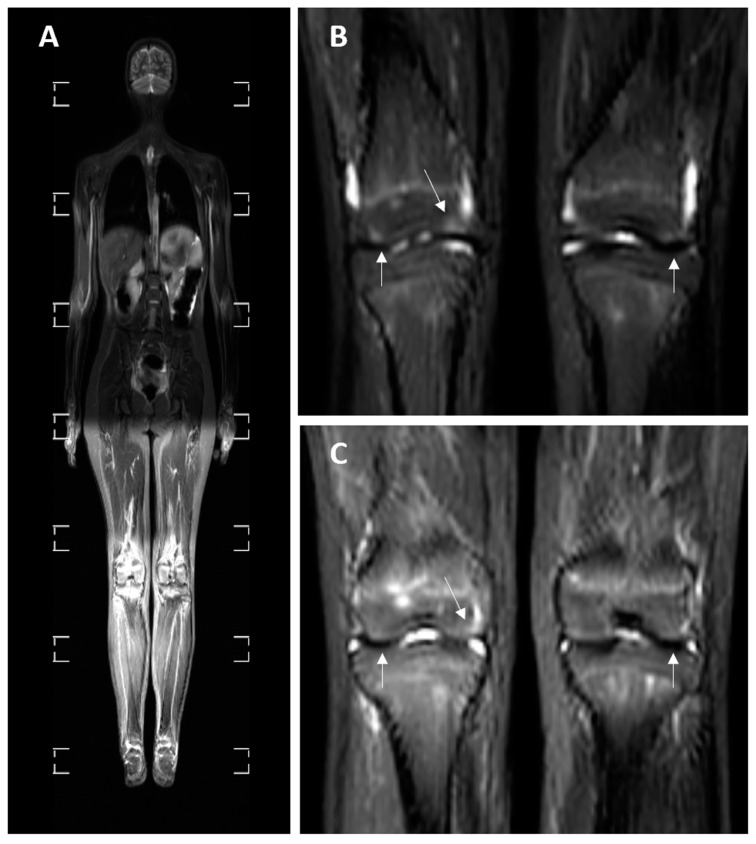
Whole-body MRI in the STIR sequence of a 14-year-old female patient with JIA and a history of knee joint pain. (**A**) A whole-body MRI scan. (**B**,**C**) Two consecutive cross-sections of the knee joints. A small amount of knee joint effusion and the signal elevation in the right femur epiphysis are notable on the images, pointed by white arrows (**B**,**C**).

Multifocal myositis was visualized on 10 (30.30%) MRI examinations (Figure 3) and was localized in the muscles of upper limbs on 4 (40%) examinations, in the muscles of lower limbs on 8 (80%) examinations, in the chest muscles on 2 (20%) examinations and in the neck muscles on 3 (30%) examinations. 

**Figure 4:**
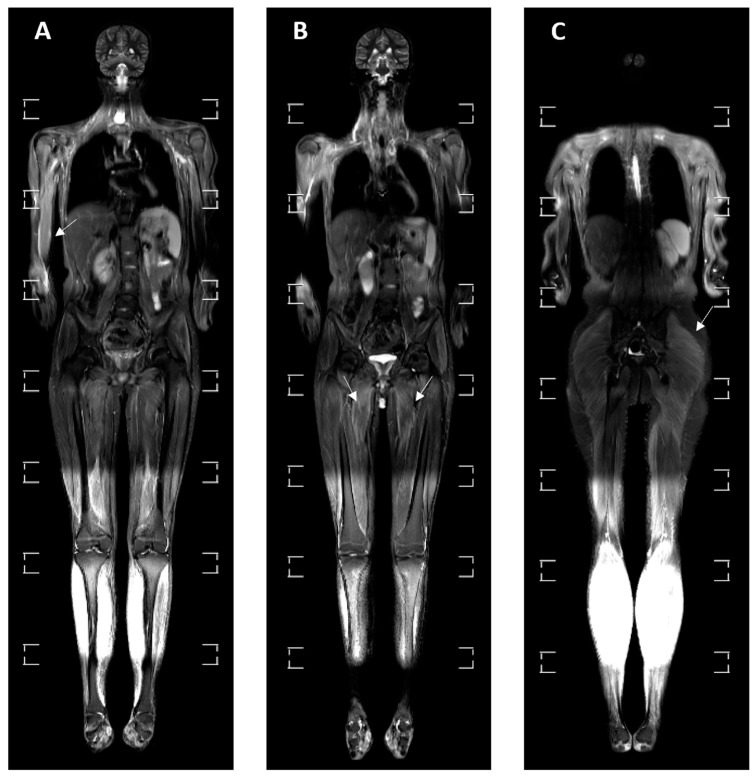
Three consecutive cross-sections (**A**–**C**) of the whole-body MRI in the STIR sequence of a 16-year-old male patient with JIIM and a history of muscle weakness. (**A**) An elevation of signal in the muscles of the right hand and forearm. (**B**) An elevation of signal in the adductor muscles of both thighs is visible, pointed by white arrows. (**C**) An elevation of signal in the left gluteus maximus muscle is notable, pointed by arrow.

Synovial fluid was visualized on nine (27.27%) MRI examinations and was localized in the joints of the upper limbs on one (11.11%) examination, in the joints of lower limbs on seven (77.78%) examinations (Figure 4) and in the sacroiliac joints on one (11.11%) examination.

Intraosseous ganglia were observed in single cases and were localized in the femur, iliac bone and calcaneus. 

In the analysis of the relationship between the occurrence of myositis or BME on MRI and the established diagnosis, there was a significant statistical relationship between myositis and the diagnosis of JIIM (*p* < 0.05). Myositis occurred statistically significantly more often in JIIM than in the other analyzed diagnoses. There were no other significant relationships between the remaining diagnosis and myositis or BME on MRI. 

## 4. Discussion 

The patients included in this study were divided into four groups in order to compare the radiological and laboratory findings with the clinical presentation of JIA, JIIM, CNO and other diseases. The most numerous group in our study were patients with a diagnosis of JIA, which is related to the fact that this is the most common rheumatic disease in children [9]. Elevated inflammatory parameters in the blood occurred in a single patient with a diagnosis of JIA. ANA was found in two patients, and HLA-B27 and HLA-Cw6 each in one patient. Synovial fluid was found in 44.44% of JIA patients and was always accompanied by pain in the joint in which it was observed. None of the patients in this group had a completely normal whole-body MRI result. This demonstrates the importance of MRI in the diagnosis of JIA, as it cannot be excluded based on low inflammatory markers or the absence of HLA-B27 or HLA-Cw6. In 85.71% of JIIM patients, the presence of myositis was detected on whole-body MRI, confirming the examination’s relevance [40]. In addition, our study demonstrated a relationship between the presence of myositis and elevated CK levels. This provides evidence that whole-body MRI accurately demonstrates muscle edema, an indication of muscle damage, of which the primary hallmark is elevated CK levels [41,42]. Whole-body MRI is the method of choice for the diagnosis of CNO [37]. In our study, in every CNO patient with confirmed BME, the lesions were multifocal and always involved the lower limbs, while in a single case, the lesions were also localized in the upper limbs. 

Whole-body MRI is not necessarily performed in every patient with a suspected rheumatic disease. This is partly because of the relatively low availability of the examination, its considerable cost and the need to sedate uncooperative children [43]. For this reason, whole-body MRI is performed in our institution mainly in children who remain challenging to diagnose and present with a vague clinical picture. This explains the two main limitations, which are the size and the heterogeneity of the population included in this study, but also highlights the role of whole-body MRI as a useful examination in so-called difficult cases. It should be noted that the time period during which 33 pediatric patients underwent whole-body MRI covered 29 months, which gives an average of about 1 patient per month. As rheumatic diseases are considered rare diseases, and additionally, as mentioned above, only a particular group of patients with suspected rheumatic diseases require a whole-body MRI, this is a significant number. 

As whole-body MRI is becoming increasingly available and will be increasingly used in clinical practice in the future, the database of patients who underwent the scan will expand. This will provide the opportunity for further analysis, including the relationship of the location of individual symptoms versus radiological findings. A study conducted at another medical center in Poland by Lanckoroński et al. [44] analyzed the whole-body MRIs of pediatric patients with CNO, JIA, their overlapping syndrome and non-specific arthropathy. The paper focused on radiological findings, and compared to our study, did not describe in detail the clinical symptoms and the results of laboratory tests. In addition, our study also included patients with JIIMs and other diseases that could be diagnosed by MRI. It is important to consider imaging studies as part of the patient’s assessment in relationship with the clinical presentation of the disease and laboratory findings in order to avoid unnecessary treatment of incidental radiological findings [45,46].

## 5. Conclusions 

This study highlights the importance of whole-body MRI, in relationship with clinical symptoms and the results of the laboratory tests, in the diagnosis of rheumatic diseases in the pediatric population. The most common radiological findings were myositis, BME and synovial fluid, while pain and limitation of joint mobility were found in more than a half of the patients. In the laboratory tests, a minority of patients had elevated inflammatory markers or positive results for ANA, HLA-B27 or HLA-Cw6. An increase in the CK level was statistically significantly related to the presence of myositis on whole-body MRI. 

## Figures and Tables

**Table 1:** Detailed information about the patients’ age. JIA—juvenile idiopathic arthritis. JIIM—juvenile idiopathic inflammatory myopathies. CNO—chronic nonbacterial osteomyelitis.

Age	All	JIA	JIIM	CNO	Others
Mean ± SD	12.73 ± 2.55	13.00 ± 2.87	12.86 ± 3.44	11.71 ± 1.70	13.10 ± 2.23
Min	8	9	8	10	10
Max	17	17	17	15	16

**Table 2:** Number of positive ANA, HLA-B27 and HLA-Cw6 results in patients (statistical significance between diagnosis was determined at *p* < 0.05 using chi-squared test).

Laboratory Test	Number of Positive Results					
	All	JIA	JIIM	CNO	Others	*p*-Value
ANA	11 (33.33%)	2 (22.22%)	6 (85.71%)	0	3 (30%)	*p* = 0.091
HLA-B27	5 (15.15%)	1 (11.11%)	1 (14.29%)	1 (14.29%)	2 (20%)	*p* = 0.886
HLA-Cw6	4 (12.12%)	1 (11.11%)	0	1 (14.29%)	2 (20%)	*p* = 0.640

**Table 3:** Number of MRI examinations, with each of the MRI findings described by the radiologist (statistical significance between diagnosis was determined at * *p* < 0.05 using chi-squared test).

MRI Findings	All	JIA	JIIM	CNO	Others	*p*-Value
BME	8 (24.24%)	2 (22.22%)	0	3 (42.86%)	3 (30%)	*p* = 0.288
Myositis	10 (30.30%)	1 (11.11%)	6 (85.71%)	1 (14.29%)	2 (20%)	*p* = 0.004 *
Synovial fluid	9 (27.27%)	4 (44.44%)	2 (28.57%)	0	3 (30%)	*p* = 0.260
Liver enlargement	1 (3.03%)	0	0	1 (14.29%)	0	*p* = 0.280
Spleen enlargement	3 (9.09%)	0	0	1 (14.29%)	2 (20%)	*p* = 0.352
Intraosseous ganglia	3 (9.09%)	2 (22.22%)	0	0	1 (10%)	*p* = 0.349

## Data Availability

The original contributions presented in the study can be directed to the corresponding author.
